# The projection from dorsal medial prefrontal cortex to basolateral amygdala promotes behaviors of negative emotion in rats

**DOI:** 10.3389/fnins.2024.1331864

**Published:** 2024-01-24

**Authors:** Youqing Cai, Jianlong Ge, Zhizhong Z. Pan

**Affiliations:** Department of Anesthesiology and Pain Medicine, The University of Texas MD Anderson Cancer Center, Houston, TX, United States

**Keywords:** optogenetics, emotion, pain, medial prefrontal cortex, basal lateral amygdala

## Abstract

Brain circuits between medial prefrontal cortex (mPFC) and amygdala have been implicated in cortical control of emotion, especially anxiety. Studies in recent years focus on differential roles of subregions of mPFC and amygdala, and reciprocal pathways between mPFC and amygdala in regulation of emotional behaviors. It has been shown that, while the projection from ventral mPFC to basomedial amygdala has an anxiolytic effect, the reciprocal projections between dorsal mPFC (dmPFC) and basolateral amygdala (BLA) are generally involved in an anxiogenic effect in various conditions with increased anxiety. However, the function of the projection from dmPFC to BLA in regulation of general emotional behaviors under normal conditions remains unclear. In this study, we used optogenetic analysis to identify how this dmPFC–BLA pathway regulates various emotional behaviors in normal rats. We found that optogenetic stimulation of the dmPFC–BLA pathway promoted a behavioral state of negative emotion, increasing anxiety-like and depressive-like behaviors and producing aversive behavior of place avoidance. Conversely, optogenetic inhibition of this pathway produced opposite effects, reducing anxiety-like and depressive-like behaviors, and inducing behaviors of place preference of reward. These findings suggest that activity of the dmPFC–BLA pathway is sufficient to drive a negative emotion state and the mPFC–amygdala circuit is tonically active in cortical regulation of emotional behaviors.

## Introduction

The medial prefrontal cortex (mPFC) regulates higher brain functions of emotion, cognition, motivation and working memory and is involved in evaluation and execution of related behaviors (Miller and Cohen, 2001; Davidson, 2002; Dalley et al., 2004). It orchestrates these brain functions through diverse efferent projections to other cortical, subcortical and thalamic regions and among them is the amygdala complex (Anastasiades and Carter, 2021; Kenwood et al., 2022). Amygdala is known as a crucial limbic structure for regulation of emotion-related behaviors including anxiety and depression of negative emotion, drug reward of positive emotion, and pain and fear of aversive behaviors (Baxter and Murray, 2002; Gottfried et al., 2003; Murray, 2007; Etkin et al., 2011; Mahan and Ressler, 2012; Fernando et al., 2013; Janak and Tye, 2015; Cai et al., 2018; Neugebauer et al., 2020). The mPFC sends strong projections to the amygdala complex and particularly to basomedial amygdala (BMA) and basolateral amygdala (BLA), which also prominently projects back to mPFC, making direct reciprocal connections between mPFC and BLA (Orsini et al., 2011; Knapska et al., 2012; Little and Carter, 2013; Burgos-Robles et al., 2017; Bloodgood et al., 2018). mPFC is mainly divided into dorsal mPFC (dmPFC) in prelimbic cortex and ventral mPFC (vmPFC) in infralimbic cortex and is composed of glutamatergic projection pyramidal neurons (PNs) and local inhibitory GABAergic interneurons (Tremblay et al., 2016; Kim et al., 2017; Ong et al., 2018; Anastasiades and Carter, 2021). The glutamatergic PNs in mPFC project to their target regions in monosynaptic pathways, forming general excitatory mPFC outputs to its projection targets (Anastasiades and Carter, 2021).

Recent studies suggest that these mPFC–amygdala projection pathways are key brain circuits in the top-down control mechanism for emotional behaviors (Shackman et al., 2011; Kenwood et al., 2022; Ressler et al., 2022). Particularly, it has been demonstrated that activation of the vmPFC–BMA pathway suppresses anxiety behavior in mice (Adhikari et al., 2015), but stimulation of the ascending BLA–mPFC pathway has an anxiogenic effect, increasing anxiety behavior (Tejeda et al., 2015; Felix-Ortiz et al., 2016; Marcus et al., 2020). Activities of the projection from dmPFC to BLA have been implicated in the increased anxiety induced by ethanol withdrawal, chronic restraint stress and chronic pain (Liu et al., 2020; McGinnis et al., 2020; Gao et al., 2023). However, stimulation of the mPFC–BLA projection blocked the anxiogenic effect of cholecystokinin infused into mPFC, indicating an anxiolytic effect of this pathway in that condition (Vialou et al., 2014). Therefore, to further characterize the normal function of the dmPFC–BLA pathway in regulation of overall emotion state, we used optogenetic analysis to identify how this specific pathway regulates typical behaviors of negative and positive emotional behaviors in normal rats.

## Materials and methods

### Animals

All procedures involving the use of animals conformed to the guidelines set by the Institutional Animal Care and Use Committee of MD Anderson Cancer Center. Wistar rats (300–400 g) of both sexes were used in this study. Our sex-based analysis showed that there was no significant sex difference, so rats of both sexes were pooled in analysis. The rats were housed in groups of three with food and water available *ad libitum*, and in a 12 h light/dark cycle. For surgery, implantation of optical fiber cannula and vector injection, a rat was anesthetized by constant inhalation of isoflurane (2%) in a stereotaxic apparatus. All behavioral trainings and tests were performed between 8: 00 am and 18: 00 pm.

### Adeno-associated viral vectors and microinjections into dorsal mPFC

Adeno-associated virus (AAV) particles of serotype 5 were obtained from the Vector Core Facility at The University of North Carolina at Chapel Hill. An AAV5-CaMKIIα-hChR2 (H134R)-mCherry vector (AAV-ChR2), an AAV5-CaMKIIα-eNpHR3.0-mCherry (AAV-eNpHR) vector or a control vector AAV5-CaMKIIα-mCherry (AAV-mCherry) was bilaterally injected (1 μL each side) into dmPFC (anteroposterior, 3.2 mm from the bregma; lateral, ±0.5 mm; ventral, −4.0 mm from dura) in rats under anesthesia in a stereotaxic instrument. Behavioral experiments were performed 4 weeks after the vector injection. After the experiments, brain tissues were harvested for anatomical identification of the injection sites. Data from injections that were outside of the targeted area were excluded.

### Implantation of optical fiber cannula and optical stimulation in BLA

Two weeks after the viral injection, a mono fiber-optic cannula (0.4 mm in diameter, Doric Lenses Inc., Canada) was stereotaxically implanted on each side of the brain just above BLA (anteroposterior, −2.8 mm from the bregma; lateral, ±5.2 mm; ventral, −7.4 mm from dura) in an anesthetized rat. After the implantation surgery, the animals were single housed and allowed to recover for 14 days before behavioral tests. For optical stimulation, the implanted cannula was connected to a 473 nm (for ChR2) or 590 nm (for eNpHR) DPSS laser (Shanghai Laser & Optic Century Co., China) through a fiber-optic patch cord with a rotary joint for free movement of the animal. For the excitatory ChR2 vector, laser light pulses of 20 Hz, 15 ms and 5 mW were delivered to BLA via the implanted cannula as we described before (Cai et al., 2018). For the inhibitory eNpHR vector, light stimulation at 1 Hz, 999-ms, 10 mW was similarly delivered. Intensity of the fiber-optic light at the end of fiber was verified before and after each experiment by a power meter (PM-100D, Thor Labs). All laser outputs were controlled by a Master-8 pulse stimulator (A.M.P.I).

### Open field test

The open field test was conducted in an illuminated chamber (72 × 72 × 50 cm) divided by a central zone and an outer zone as we described before (Cai et al., 2018; Ge et al., 2022). A rat was connected to the light source and was placed in the center of the chamber. The rat was allowed to move freely for 15 min and locomotion activity of the animal in the two zones was video-recorded and analyzed with an automated video-tracking system (EthoVision XT, Noldus Information Technology Inc.). In the test, a total test time of 15 min was divided into three consecutive 5-min periods with the light off in the 1st period (control), light on in the 2nd period and light off in the 3rd period in rats with dmPFC injection of excitatory vector AAV-ChR2 or inhibitory vector AAV-eNpHR, and corresponding control vector AAV-mCherry. Reduced time spent and distance traveled in the unprotected central zone (central time and central distance, respectively) were regarded as anxiety-related indices. The total distance traveled in the entire chamber during the test was recorded and used as a measure of general locomotor activity.

### Forced swim test

The forced swim test was conducted in a cylinder (diameter 30.5 × height 45.7 cm) for rats (ENV-590R, Med Associates Inc.) according to the protocol described in previous reports (Slattery and Cryan, 2012; Cai et al., 2018). On day 1, a rat was placed into the water-filled cylinder for 15-min for pre-test swim. On day 2, the rat was connected with the light source and was allowed to swim for 5 min. Light was delivered for 1-min before and 5-min during the swim test in the AAV-ChR2-, AAV-eNpHR-, or AAV-mCherry-injected rats. The swim activity was videotaped and immobility time was counted manually afterwards. Immobility was defined as cessation of active swimming and escaping activities. Time the animal spent immobile during the test was recorded as a measure of despair-like behavior.

### Test of conditioned place preference and conditioned place aversion

Detailed procedures of conditioned place preference (CPP) and conditioned place aversion (CPA) have been described in our previous studies (Zhu et al., 2007; Bie et al., 2009; Hou et al., 2015; Cai et al., 2018). CPP and CPA tests were conducted in a standard 3-chamber CPP apparatus (MED Associates, St. Albans, VT). The rats were subjected to 2 sessions of habituation, one session per day for 2 days before the CPP/CPA test. In a habituation session, a rat was placed in the center connecting chamber and allowed to move freely between the two test chambers for 30 min. After habituation to the test chambers, a rat was placed in the center chamber and was allowed to move freely among the chambers for 15 min in a pre-test. The time the rat spent in each chamber was recorded automatically. Then, the rat received 4 conditioning sessions for 8 days, each two-day session consisting of light stimulation-pairing conditioning for 30 min in a chamber on day 1 and no light stimulation-pairing conditioning for 30 min in the other chamber on day 2. After the four conditioning sessions, the rat underwent a post-test for 15 min with the same procedures as in the pre-test. The AAV-ChR2-, AAV-eNpHR- or AAV-mCherry-injected rats were subject to the same conditioning procedures. The CPP/CPA score was defined as the difference in time the rat spent in the light-paired chamber between the pre-test and the post-test for the same rat.

### Tests for thermal and mechanical pain

For thermal pain, a rat was placed in a Plantar Test Instrument (Model 37,370, Ugo Basile, Italy). Paw withdrawal response to an infrared heat stimulus was measured with a Hargreaves apparatus. Latency from the onset of the heat stimulus to the paw-withdrawal response was recorded automatically by the apparatus and was measured twice with a 5-min interval. The data presented were the averaged values of paw-withdrawal latencies of both right and left hind paws measured alternatively. For mechanical pain, a rat was extensively handled and habituated to the test environment and test apparatus for 3 d before the pain test. Then, the rat was placed in a plastic box with mesh floor and allowed to acclimate for 20 min. A series of calibrated von Frey filaments were applied perpendicularly to the plantar surface of a hind paw with sufficient force to bend the filament for 6 s. A brisk movement of the hind paw (withdrawal or flinching) was considered as a positive response. The threshold (g) of the tactile stimulus producing a 50% likelihood of withdrawal was determined by the “up-down” calculating method (Chaplan et al., 1994). The hind paw withdrawal response was measured twice with a 5-min interval. The latency and the threshold were measured before optical stimulation as baseline control and after 5-min optical stimulation administered 2–3 min after completion of baseline measurements in AAV-ChR2-, AAV-eNpHR- or AAV-mCherry-injected rats.

### Novel object recognition assay for non-spatial memory

Novel object recognition assay is a well-established method to evaluate non-spatial memory in an open field (Bevins and Besheer, 2006). As we showed previously (Cai et al., 2013), the test includes 3-sessions in 2 days for habituation, training and retention test. On day 1, rats were habituated individually in a grey open arena (60 × 60 × 50 cm for L × W × H) for approximately 10 min without any object in the field. Two objects placed in the opposite corners of the arena were used in the test: one was a yellow glass cylinder (H = 12 cm, *r* = 3 cm) and the other was a green plastic cuboid (H = 12, L = 6, W = 3.7 cm), and both had similar surface area and the same height. For the training session on day 2, two identical objects were placed in the arena. The rat was allowed to freely explore the objects for 5 min and the amount of time the animal explored on each object was videotaped for analysis afterwards. For the retention test 4 h after the training session, one object from the training session (familiar object) was randomly replaced by a novel object. The rat was allowed to freely explore the objects in the field for 5 min. The time spent exploring on each object was manually counted from the recorded videos. The preference ratio for novel object was determined by the time exploring on the novel object/total test time (5 min).

To minimize the potential influence of the same and different behavioral tests above on the same animals, we conducted the same test only once, reversed the order of different tests (e.g., OFT and FST), and waited at least 3 days between the two different tests in the same animals.

### Immunohistochemistry

A rat was deeply anesthetized with pentobarbital and transcardially perfused with heparinized saline and subsequently with ice-cold 4% paraformaldehyde in 1 × PBS (pH 7.4). The brain was removed and post-fixed in 4% paraformaldehyde overnight at 4°C, followed by dehydration with 30% sucrose in 1 × PBS. Tissues were sectioned into 30-μm thick coronal sections with a cryostat at-20°C. Sections were blocked with 5% normal donkey serum in PBS containing 0.3% Triton X-100 and incubated overnight with primary antibodies (mouse or rabbit anti-mCherry antibodies from Abcam, ab167453 or ab125096, 1: 500 dilution). Sections were then rinsed and incubated with the Alexa Fluor-conjugated secondary antibodies (1: 500, Alexa Fluor 488, green color, or 568, red color, Invitrogen), and were mounted on slides, dried and cover-slipped with ProLong Gold anti-fade reagent. The stained sections were examined with an Olympus BX51 fluorescence microscope or a Zeiss 710 confocal microscope.

### Statistical analysis

Comparisons of averages of two groups were performed with the unpaired, two-tailed Student’s t test. Two-way ANOVA for repeated measures with *post hoc* analysis of the Bonferroni method was used to determine statistical significance in behavioral experiments for effects of treatment and between-group interactions at each time point. Data were tested with the Shapiro–Wilk test for normal distribution. All data sets passed the normality test (*p* > 0.05), suggesting a normal distribution of the data. A *p* < 0.05 was considered statistically significant. All statistical analyses were performed with the Prism software version 9.0 (GraphPad Software). Data are presented as mean ± S.D.

## Results

### Dorsal mPFC excitatory neurons project to BLA, not to CeA

**Figure 1:**
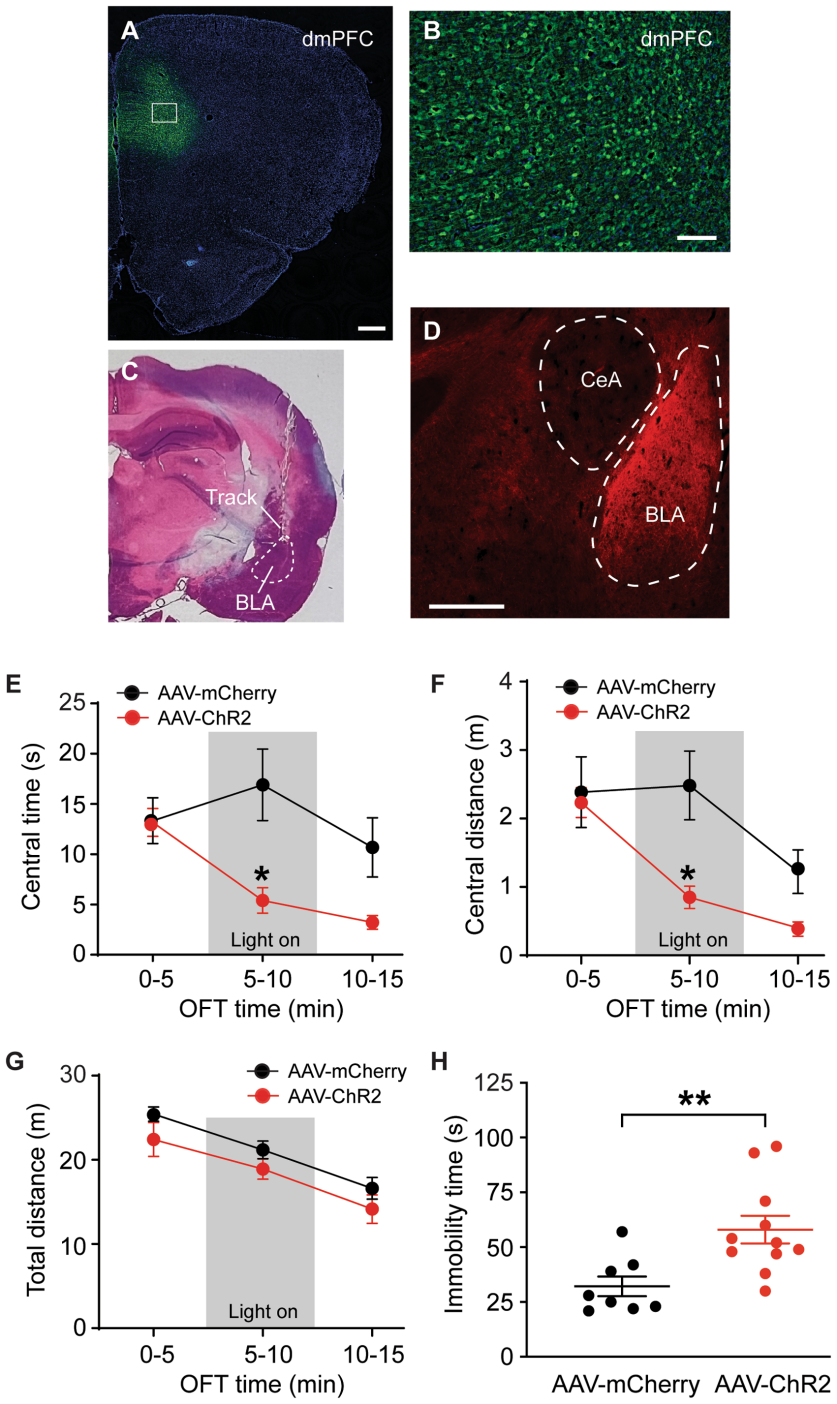
Stimulation of dmPFC–BLA projections induces behaviors of negative emotion. **(A,B)** Immunohistochemical images of ChR2-mCherry expression in dorsal medial prefrontal cortex (dmPFC, **A**) and with high magnification in the dmPFC area as marked in A **(B)** 4 weeks after bilateral infusion of the viral vector AAV5-CaMKIIα-ChR2-mCherry (AAV-ChR2-mCherry) into the mPFC of a rat. **(C)** A representative brain section stained by hematoxylin eosin, showing a cannula track targeting the basolateral amygdala (BLA). **(D)** An immunohistochemical image showing ChR2-mCherry expression in BLA, but not in the central nucleus of the amygdala (CeA) 4 weeks after similar infusion of the AAV-ChR2-mCherry vector into the dmPFC in a rat. Secondary antibody Alex-Fluor 488 (green) was used in **A** and **B**, and Alex-Fluor 568 (red) was used in **D**. Scale bars = 500 μM **(A,D)** and 50 μM **(B)**. **(E–G)** Group data of time spent **(E)** and distance traveled **(F)** in central zone and total distance traveled **(G)** in rats with mPFC injection of the control vector AAV-mCherry (*n* = 6) and AAV-ChR2 vector (*n* = 6) in three consecutive 5-min periods in the open field test. The light was on during the 2nd period (grey areas) for optical stimulation of the mPFC–BLA projections. **(H)** Immobility time in rats with mPFC injection of the control vector (*n* = 8) and AAV-ChR2 vector (*n* = 11) during optical stimulation in BLA in the forced swim test. Errors are S.E.M in all figures. ^*^p < 0.05, ^**^p < 0.01.

We used a viral vector and immunomicroscopy to identify axon projections of excitatory PNs in dmPFC to amygdala in rats. The vector AAV-CaMKII-hChR2-mCherry was bilaterally injected into dmPFC of naïve rats to transfect local excitatory (CaMKIIα-expressing) PNs and their axon terminals in their projection areas. Four weeks later, we examined mCherry expression in the dmPFC and in the amygdala. Intense mCherry staining was observed in the vector-infused dmPFC, suggesting successful transfection of local excitatory PNs with the vector through CaMKIIα promoters (Figures 1A,B). Figure 1C shows a representative image of the brain section stained by hematoxylin eosin with a cannula track targeting BLA. In the amygdala, we found robust mCherry expression in BLA, but not in the central nucleus of amygdala (CeA) (Figure 1D). This result illustrates selective and strong excitatory projections from dmPFC to BLA, but not to CeA.

### Stimulation of dmPFC–BLA projections induces anxiety- and depressive-like behaviors

We examined the function of this dmPFC–BLA pathway by optogenetic activation of this pathway and real-time behavioral tests of anxiety-like and depressive-like behaviors of negative emotion in naïve rats *in vivo*. Four weeks after bilateral infusion of the AAV-CaMKII-hChR2-mCherry vector (AAV-ChR2, *n* = 6) or a control vector AAV-CaMKII-mCherry (AAV-mCherry, *n* = 6) into the rat dmPFC, we found that the AAV-ChR2- and AAV-mCherry-injected rats displayed a similar level of anxiety-like behavior as measured by the open field test (OFT) during the initial 5-min period without stimulation (light off) (time spent in central zone or central time: control, 13.3 ± 5.4 s, ChR2, 13.2 ± 3.4 s, *t* = 0.052, *p* > 0.99, multiple comparisons of 2-way ANOVA). However, in the 5-min period immediately following the preceding period, with optical stimulation (light on) in the BLA to activate the dmPFC–BLA pathway, the AAV-ChR2-injected rats showed significantly decreased central time indicating increased anxiety behavior when compared to the control rats (central time with light: control, 16.9 ± 8.8 s, ChR2, 5.4 ± 2.9 s, *F*_(2,30)_ = 4.0 and *p* = 0.028, *t* = 3.60 and *p* < 0.05, multiple comparisons of 2-way ANOVA, Figure 1E). During the following 5-min period without stimulation (light off), the central time was no longer statistically different between the ChR2-injected and control animals (central time: control, 10.7 ± 7.3 s, ChR2, 3.2 ± 1.7 s, *t* = 2.34, *p* = 0.078, multiple comparisons of 2-way ANOVA). Similar results were obtained in the distance traveled by the rats in the central zone (Figure 1F). In contrast, the total distance travelled in the entire test chamber during each of the three periods was not different between the two rat groups, indicating that the optical stimulation did not affect the overall locomotor activity of the rats (Figure 1G). In addition, we determined the effect of activating this dmPFC–BLA pathway on the depressive-like behavior of another form of negative emotion by the forced swim test (FST). In mostly the same two groups of rats injected with AAV-ChR2 (*n* = 11) or AAV-mCherry (*n* = 8), we found that optical stimulation of the dmPFC-BLA pathway significantly increased immobility time indicating depressive-like behavior in the FST (control, 32.1 ± 12.7 s, ChR2, 58.0 ± 20.9 s, *t* = 3.09, *p* = 0.006, Figure 1H). These findings suggests that acute activation of the excitatory dmPFC-BLA pathway is sufficient to induce anxiety-like and depressive-like behaviors of negative emotion in rats under normal condition.

### Stimulation of dmPFC-BLA projections induces behaviors of aversion and memory impairment

**Figure 2:**
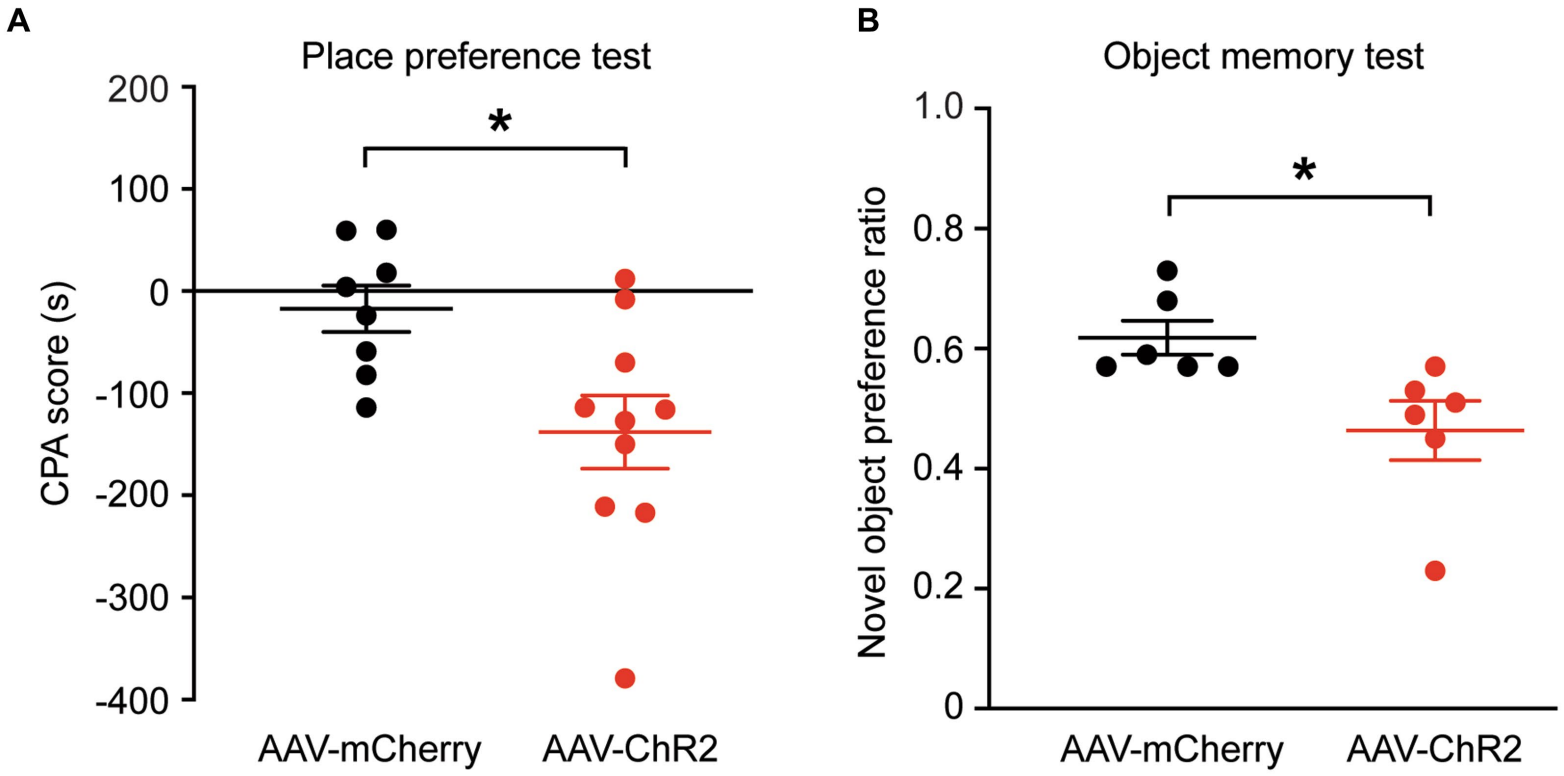
Stimulation of dmPFC-BLA projections induces behaviors of aversion and memory impairment. **(A)** Scores of conditioned place aversion (CPA) in rats with dmPFC injection of the control vector (*n* = 8) and AAV-ChR2 vector (*n* = 10) after conditioning sessions paired with the optical stimulation in BLA. **(B)** Preference ratios for novel object in rats (*n* = 6 each group) after dmPFC injection of the AAV-mCherry or AAV-ChR2 vector in the novel object recognition test. ^*^p < 0.05.

We then determined whether stimulation of the dmPFC-BLA projection would induce aversive behavior, another form of negative emotion, with the conditioned place aversion (CPA) test. Separate groups of rats with AAV-ChR2 or AAV-mCherry injection into dmPFC were conditioned with the optical stimulation in BLA. After the 4 conditioning sessions for 8 days, we found that optical stimulation of the dmPFC-BLA projection produced strong place aversion in the ChR2-injected rats (*n* = 10), but not in the control rats (*n* = 8), as measured by the CPA score (control, −17.2 ± 63.9 s, ChR2, −138.0 ± 112.9 s, *t* = 2.69, *p* = 0.02, Figure 2A). Thus, it appears that activating the dmPFC-BLA projection can also induce strong place aversion of negative reinforcement in normal rats.

Next, we wondered whether these emotional changes could affect memory. We tested the recognition memory using the novel object recognition test in the same groups of rats. We found that, after optical stimulation in the BLA, the AAV-ChR2-injected rats (*n* = 6) spent significantly less time on the novel object than the control vector-injected rats (*n* = 6), as measured by the preference ratio for novel object (control, 0.62 ± 0.07, ChR2, 0.46 ± 0.12, *t* = 2.72, *p* = 0.0216, Figure 2B). These results show that activation of the excitatory dmPFC-BLA pathway likely attenuates the recognition memory for novel object as measured in this test.

### Inhibition of dmPFC–BLA projections reduces anxiety- and depressive-like behaviors

**Figure 3:**
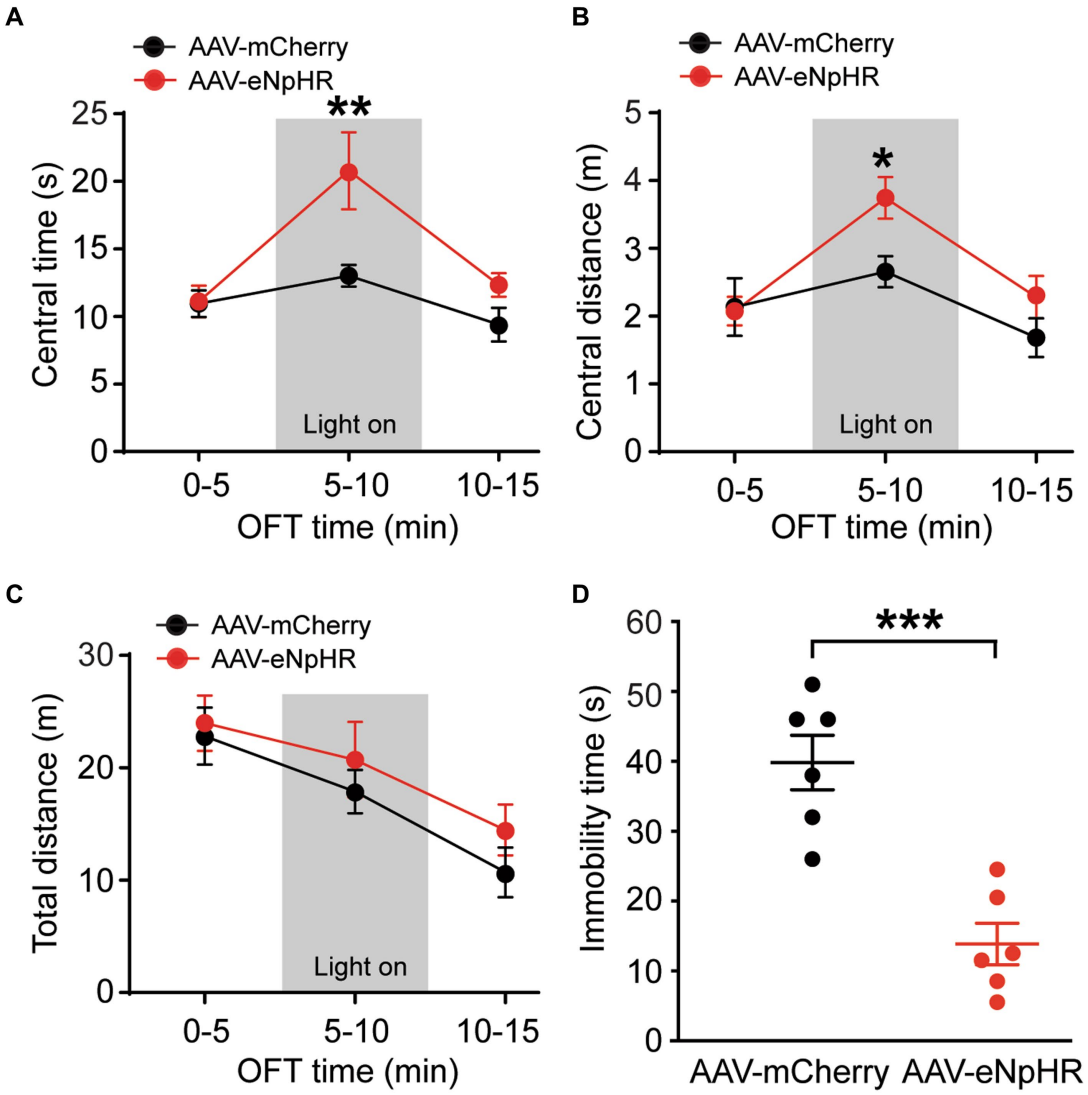
Inhibition of dmPFC–BLA projections reduces behaviors of negative emotion. **(A–C)** Group data of time spent **(A)** and distance traveled **(B)** in central zone and total distance traveled **(C)** in rats with mPFC injection of the control vector AAV-mCherry (*n* = 6) and the inhibitory vector AAV-eNpHR (*n* = 7) in three consecutive 5-min periods in the open field test. The light was on during the 2nd period (grey areas) for optical inhibition of the dmPFC–BLA projections. **(D)** Immobility time in rats with dmPFC injection of the control vector (*n* = 6) and AAV-eNpHR vector (*n* = 6) during optical stimulation in BLA in the forced swim test. ^*^p < 0.05, ^**^p < 0.01, ^***^p < 0.001.

To further validate the functions of this dmPFC–BLA pathway, we optically inhibited the pathway and investigated its effects on the same emotion-related behaviors in naïve rats. The inhibitory vector AAV-CaMKII-eNpHR3.0-mCherry (AAV-eNpHR, *n* = 7) or the control vector AAV-mCherry (*n* = 6) was bilaterally injected into dmPFC and 4 weeks later, optical stimulation was administered during the behavioral tests. We found that, while the two groups of rats had no difference in the central time without light stimulation in the first 5-min period in the OFT (central time: control, 10.9 ± 2.4 s, AAV-eNpHR, 11.1 ± 3.2 s, *t* = 0.07, *p* > 0.99, multiple comparisons of 2-way ANOVA), the AAV-eNpHR-injected animals displayed significantly increased central time indicating decreased anxiety-like behavior during the following 5-min period with optical stimulation (central time with light: control, 13.0 ± 2.0 s, AAV-eNpHR, 20.8 ± 7.4 s, *F*_(1,11)_ = 10.56 and *p* = 0.0006, *t* = 3.50 and *p* = 0.004, multiple comparisons of 2-way ANOVA, Figure 3A). During the following 5-min period without light stimulation, there was no difference in central time between the two rat groups (central time: control, 9.4 ± 2.9 s, AAV-eNpHR, 12.3 ± 2.4 s, *t* = 1.32, *p* = 0.59, multiple comparisons of 2-way ANOVA). The central distance was also significantly increased in the AAV-eNpHR-injected rats during the optical stimulation period (Figure 3B). The total distance traveled for overall locomotor activity during each of the three 5-min periods had no difference between the two rat groups (Figure 3C). We then examined the depressive-like behavior after the optical inhibition of the dmPFC–BLA pathway in these two groups of rats with the FST. We found that, when compared with the control vector-injected rats (*n* = 6), light stimulation in the AAV-eNpHR-injected rats (*n* = 6) significantly reduced immobility time, consistent with decreased depressive-like behavior in the FST (control, 39.8 ± 9.6 s, AAV-eNpHR, 14.3 ± 7.3 s, *t* = 5.205, *p* = 0.0004, Figure 3D).

These results demonstrate that selective optical inhibition of the excitatory dmPFC-BLA projection attenuates anxiety-like and depressive-like behaviors, just opposite to the effects of activating this pathway shown earlier, further supporting the notion that activation of the excitatory dmPFC-BLA pathway promotes behaviors of negative emotion with anxiogenic and depressive effects in rats under normal conditions.

### Inhibition of dmPFC-BLA projections promotes behaviors of place preference and memory

**Figure 4:**
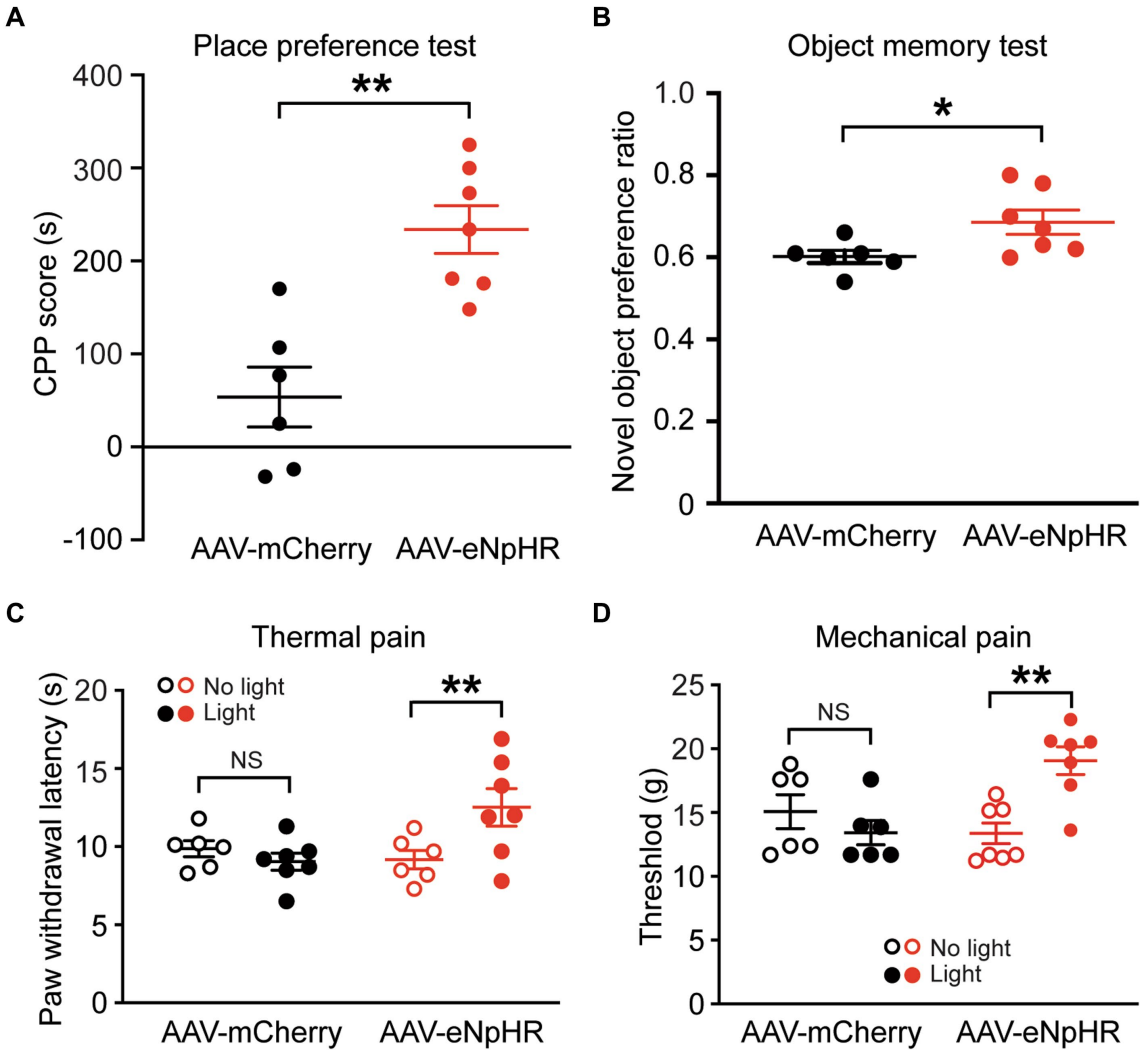
Inhibition of dmPFC-BLA projections facilitates behaviors of reward and memory and reduces nociceptive responses. **(A)** Scores of conditioned place preference (CPP) in rats with dmPFC injection of the control vector (*n* = 6) and the inhibitory AAV-eNpHR vector (*n* = 7) after conditioning sessions paired with the optical stimulation in BLA. **(B)** Preference ratios for novel object in rats after dmPFC injection of the AAV-mCherry (*n* = 6) or AAV-ChR2 vector (*n* = 7) in the novel object recognition test. **(C,D)** Paw-withdrawal latencies for thermal pain **(C)** and paw-withdrawal thresholds for mechanical pain **(D)** before (no light) and after (light) optical stimulation in the BLA for inhibition of the dmPFC–BLA projections in rats with dmPFC injection of the control vector AAV-mCherry (*n* = 6) or the inhibitory vector AAV-eNpHR (*n* = 7). NS, not significant. ^*^p < 0.05, ^**^p < 0.01.

We further tested the effects of inhibiting the dmPFC-BLA projection on reward-and memory-related behaviors. In separate groups of naïve rats injected with AAV-mCherry or AAV-eNpHR in the dmPFC, we first examined behavior of conditioned place preference (CPP) related to a reward effect. After conditioning the rats with optical stimulation in the BLA, we found that light stimulation in the AAV-eNpHR-injected rats (*n* = 7) produced robust CPP behavior of reward when compared with the AAV-mCherry-injected control rats (*n* = 6) (CPP score: control, 53.8 ± 79.1 s, AAV-eNpHR, 233.9 ± 68.0 s, *t* = 4.424, *p* = 0.001, Figure 4A). We then determined how inhibiting the dmPFC-BLA pathway would affect memory-related behavior with the novel object recognition test. In the same two groups of rats, we found that, after the optical stimulation in the BLA, the AAV-eNpHR group (*n* = 7) displayed higher preference for the novel object than the control group (*n* = 6) (preference ratio: control, 0.60 ± 0.05, AAV-eNpHR, 0.69 ± 0.08, *t* = 2.37, *p* = 0.037, Figure 4B).

These results suggest that inhibiting the dmPFC-BLA pathway induces a rewarding effect and likely promotes recognition memory, again opposite to the effects of activating this pathway as shown earlier. Thus, it appears that the excitatory dmPFC-BLA projection, once activated, reduces reward-and memory-related behaviors under normal conditions.

### Inhibition of dmPFC-BLA projections reduces nociceptive response

Finally, we determined how inhibiting the dmPFC-BLA projection would change pain response, the behavior often associated with negative emotion. In separate groups of naive rats injected with the AAV-eNpHR vector (*n* = 7) or the AAV-mCherry vector (*n* = 6) into the dmPFC, we found that the light stimulation in the BLA significantly reduced nociceptive responses both to thermal stimulus and to mechanical stimulus with increased paw withdrawal latency in the thermal pain test (AAV-mCherry: before stimulation, 9.8 ± 1.2 s, after stimulation, 9.2 ± 1.5 s, *t* = 0.818, *p* = 0.862; AAV-eNpHR: before stimulation, 9.0 ± 1.3 s, after stimulation, 12.5 ± 3.2 s, *F*_(1,11)_ = 5.647 and *p* = 0.037, *t* = 4.382 and *p* = 0.002, multiple comparisons of 2-way ANOVA, Figure 4C), and increased paw-withdrawal threshold in the von Frey test (AAV-mCherry: before stimulation, 15.1 ± 3.2 g, after stimulation, 13.4 ± 2.2 g, *t* = 1.108, *p* = 0.583; AAV-eNpHR: before stimulation, 13.01 ± 2.1 g, after stimulation, 19.1 ± 2.9 g, *F*_(1,11)_ = 13.02 and *p* = 0.004, *t* = 4.114 and *p* = 0.003, multiple comparisons of 2-way ANOVA, Figure 4D). These findings indicate that inhibiting this dmPFC–BLA pathway can also reduce nociceptive responses.

## Discussion

In this study, we have shown that activation of the specific pathway from dmPFC to BLA is sufficient to cause a series of behaviors of negative emotion including anxiety-like and depressive-like behaviors, and place aversion with likely impaired recognition memory. Our results of inhibiting this pathway suggest that this pathway is tonically active in cortical regulation of these behaviors of negative emotion under normal conditions. Together, these findings suggest that activity of this dmPFC–BLA pathway can sufficiently drive a behavioral state of negative emotion in rats.

Anxiety has been the research focus in recent studies on the functions of specific pathways for the reciprocal connections between mPFC and amygdala. Using optogenetic stimulation, Adhikari et al. have shown that neurons in vmPFC mainly target BMA and stimulation of the vmPFC–BMA pathway decreases anxiety in mice (Adhikari et al., 2015). In contrast, studies on the reciprocal projections between dmPFC and BLA generally suggest an anxiogenic effect of the dmPFC–BLA circuits. Particularly for the mPFC–BLA projection, it has been shown that activity of this descending projection contributes to the increased anxiety in various anxiety-related animal models including ethanol withdrawal, stress, chronic pain and fear (Cho et al., 2013; Liu et al., 2020; McGinnis et al., 2020; Gao et al., 2023; Gunduz-Cinar et al., 2023). Our current study shows that the activity of the dmPFC–BLA pathway tonically maintains an emotional status as part of top-down cortical control of emotional behaviors under normal conditions, as its activation is sufficient to drive towards a more negative emotional state and its inhibition causes the shift to a more positive emotional state involving a series of emotion-related behaviors including anxiety-like and depressive-like behaviors, place preference of reward, aversive pain responses, and recognition memory.

Previous studies have shown that dmPFC and vmPFC have differential roles in regulation of other emotional behaviors including fear, drug seeking and reward (Ishikawa et al., 2008; LaLumiere and Kalivas, 2008; Sangha et al., 2014; Gourley and Taylor, 2016; Trask et al., 2017; Caballero et al., 2019). For fear regulation, in particular, activity in dmPFC inputs to lateral amygdala neurons promotes fear expression while the pathway of vmPFC to basal and basolateral amygdala decreases fear (Maren and Quirk, 2004; Herry et al., 2010; Orsini et al., 2011; Sierra-Mercado et al., 2011; Knapska et al., 2012). Thus, the promoting role of the dmPFC–lateral amygdala in fear, another form of negative emotion, is consistent with our current results showing that activating the dmPFC–BLA pathway induces anxiety-like and depressive-like behaviors, and place aversion of negative emotion. Thus, it appears that vmPFC that mainly projects to BMA and dmPFC that selectively projects to BLA have opposing effects in regulation of anxiety and other emotional behaviors, providing a bi-directional cortical control of emotion through amygdala.

Pain is an aversive experience involving several forms of negative emotion such as anxiety and depression (Wilson et al., 2001; McWilliams et al., 2003; Wiech and Tracey, 2009). mPFC has been implicated in regulation of pain, but the detailed neuronal pathways and neural mechanisms involved remain poorly understood (Ong et al., 2018). Our current results show that inhibiting the dmPFC–BLA pathway reduces pain responses, which is consistent with its effects on other emotional behaviors in promoting a positive emotional state. It also indicates that tonic activity of this pathway maintains pain sensitivity under normal conditions.

It is interesting to observe in our results that changing the activity of the dmPFC–BLA pathway alters the memory involved in novel object recognition memory, but not CPA behavior that probably also involves memory of environment. This is likely due to the striking and distinct properties and characteristics in object recognition-involved memory and CPA-involved memory. CPA memory is strongly emotional and aversive, induced by an aversive and external stimulus, and is intense in degree and long lasting (Hou et al., 2015), whereas object recognition memory is emotionally neutral, occurs naturally without manipulating stimulus, and is much more subtle and acute. Given these differences, our results may suggest that the dmPFC–BLA pathway could alter the memory related to novel object learning and memory, but not the memory induced by strong aversive stimulation, or the latter memory is simply so intense that it overwhelms any effect by manipulating activity of the dmPFC–BLA pathway in the behavioral test under our experimental settings.

In summary, findings from this study suggest that activity of the dmPFC–BLA pathway is sufficient to drive and promote a state of negative emotion under normal conditions in a tonically active way in the circuit mechanisms for a bi-directional mPFC control of emotion.

## Data availability statement

The raw data supporting the conclusions of this article will be made available by the authors, without undue reservation.

## Ethics statement

The animal study was approved by Animal Care and Use Committee of University of Texas MD Anderson Cancer Center. The study was conducted in accordance with the local legislation and institutional requirements.

## Author contributions

YQC: Conceptualization, Data curation, Formal analysis, Investigation, Methodology, Project administration, Validation, Writing – original draft, Writing – review & editing. JG: Data curation, Formal analysis, Investigation, Methodology, Validation, Writing – review & editing. ZZP: Conceptualization, Data curation, Formal analysis, Funding acquisition, Investigation, Methodology, Project administration, Resources, Supervision, Validation, Writing – original draft, Writing – review & editing.
